# EVALUATING THE RELATIONSHIP BETWEEN MANDIBULAR PLANE AND LOWER OCCLUSAL PLANE IN FULLY DENTULOUS PATIENTS

**DOI:** 10.12688/f1000research.179633.1

**Published:** 2026-05-12

**Authors:** Mallikarjuna Ragher, Sanath Kumar Shetty, Vidya Bhat, Rajesh Shetty, Savitha Dandekeri, Sanha Razdan, Nafiya Abdul Aziz

**Affiliations:** 1 PROSTHODONTICS, Yenepoya (Deemed to be University) Dental College, Mangaluru, Karnataka, India

**Keywords:** Cephalometric Landmarks, Fox Bite Plane, Frankfort’s Horizontal Plane, Occlusal Plane

## Abstract

**Purpose:**

The inclination of the occlusal plane is important for diagnostic purposes, and it also acts as a guideline for designing occlusal rehabilitation therapies. When all teeth are lost, different landmarks have been employed to establish the occlusal plane. Hence, this study aims to compare the mandibular plane with the lower occlusal plane in dentulous patients.

**Methods:**

A digital photograph of the right and left facial profiles was obtained of the angle between the intraoral occlusal plane (represented by a modified Fox plane) and mandibular plane (represented by a metallic scale placed on the lower border of the mandible) in a standard position. The angle was calculated using Microdicom software, and results were statistically analysed using Mann Whitney U test.

**Results:**

The average angle between the occlusal plane and mandibular plane was 6 degrees and the difference in angle between the right and left sides was not statistically significant.

**Conclusions:**

The lower occlusal and mandibular plane though not parallel to each other clinically, may serve as an initial guide in the establishment of the occlusal plane.

## Introduction

According to *The Glossary of Prosthodontics Terms*, an occlusal plane has been defined as ‘the average plane established by the incisal and occlusal surfaces of the teeth. Generally, it is not a plane but represents the planar mean of the curvature of these surfaces. ^1^

The inclination of the occlusal plane (IOP) is an important factor that determines occlusal balance. ^2^

The most crucial factor that governs the balance in occlusion is the inclination of the occlusal plane. In dentulous and edentulous patients, the occlusal plane is important for diagnostic purposes and also acts as a base for designing rehabilitation therapies. ^3^

Occlusal plane analysis is required in various clinical situations like full mouth rehabilitation, complete denture fabrication, plane correction, etc. In dentate individuals, the IOP is generally compared with the Frankfort plane (FP) (porion-orbitale).

The various other intraoral landmarks used to establish the occlusal plane are the retromolar pad, the lateral borders of the tongue, the position of the parotid papilla, the commissures of the lips, and buccinator grooves. The most used landmark is the retromolar pad, which is difficult to locate in dentate individuals with 2 ^nd^ molars.

Another method to locate the occlusal plane is by using the line passing from the ala of the nose to the external auditory meatus or tragus. There is no consensus on the point of the tragus as it varies among population, gender, and other factors. ^4^

Radiographic landmarks lead to the exposure to unnecessary radiation to the patients; hence, they cannot always be recommended.

An occlusal plane analyser has been used to aid in developing an initial mandibular occlusal plane in diagnosis and treatment plan and later as an important element of both developing the contours of definitive restorations and as a guide for actual tooth preparation. ^5– 6^

The Broadrick Occlusal Plane Analyser, that is the Broadrick flag, permits reconstruction of the curve of Spee in harmony with the anterior and condylar guidance. It assists in locating the cusp tips of the posterior teeth. ^6^

The accuracy of the Broadrick plane is also questionable in subjects with attrition or in cases where there is the absence of mandibular canine and 2 ^nd^ molar. Hence, other methods could be used with this method to determine the IOP accurately.

Hence, this study evaluates whether the mandibular plane is parallel to the occlusal plane in dentulous subjects.

## Materials and methods

The sample size was calculated using G-Power software with the level of significance at 1% level of significance and a standard deviation of 2.03 with a 1% margin of error, the minimum sample size required was 97; hence, the total sample size taken for this study was 100.

Ethical approval was obtained from Yenepoya Ethics Committee-2, Yenepoya (Deemed to be University) (YEC2/636), and 100 volunteers of 25–30 years of age meeting the inclusion/exclusion criteria were enrolled for the study. The proposed research was explained to them, and written informed consent was obtained.

The inclusion criteria included:

1. Straight or orthognathic profile.
2. No previous history of orthodontic treatment.
3. No congenitally missing or extracted teeth.
4. Well-formed occlusion.

Participants with class 2 and 3 malocclusions were not considered in the study.

**Figure 1.**
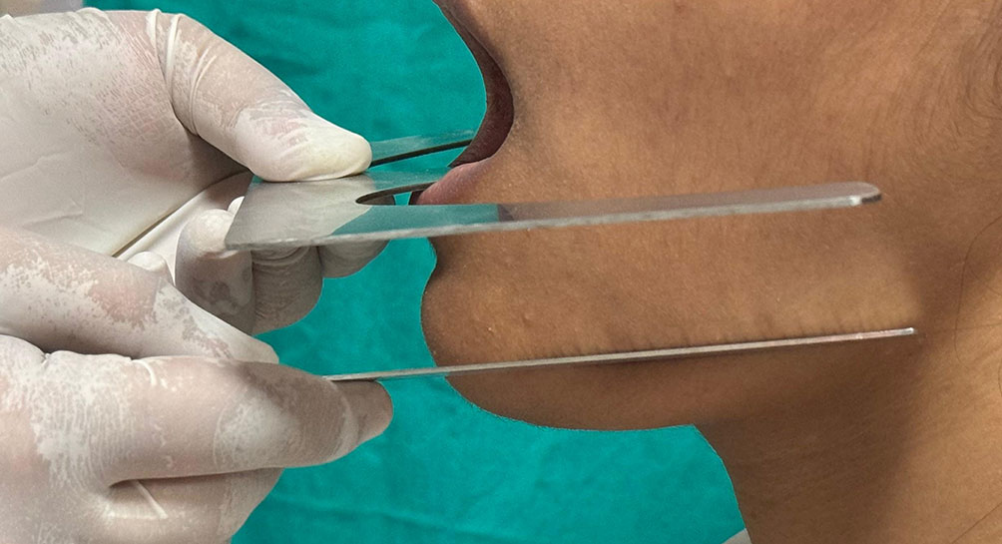
Occlusal and mandibular plane on the left side.

**Figure 2.**
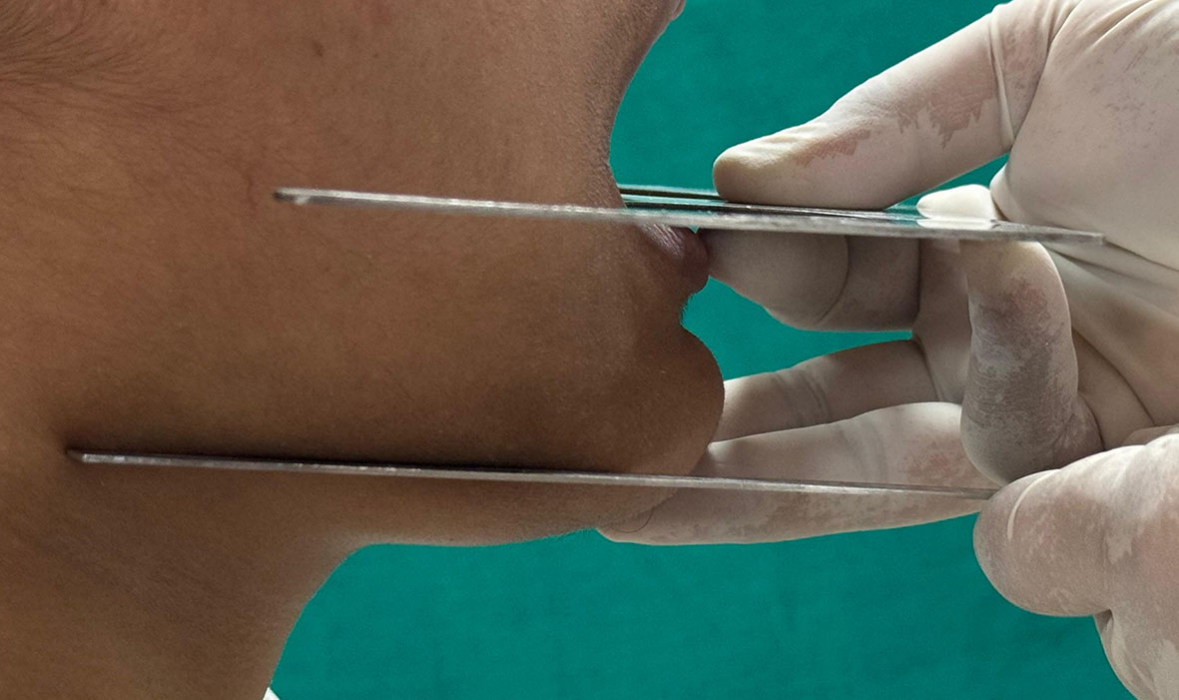
Occlusal and mandibular plane on the right side.

Participants were asked to sit in a comfortable upright position. A metal scale was placed on the lower border of the mandible, contacting the angle of the mandible posteriorly and the border of the chin anteriorly, representing the mandibular plane. A modified Trubyte occlusal plane plate (Fox Bite plane) was positioned intraorally to the distobuccal cusp of the last molar and the tip of the canine, which represents the lower occlusal plane. The outer wing of the plate indicates the position of the lower occlusal plane extraorally, as shown in Figures 1 and 2. A digital photograph of the facial profile was taken using an SLR digital camera.

The photograph was standardized according to the following criteria.

**Figure 3.**
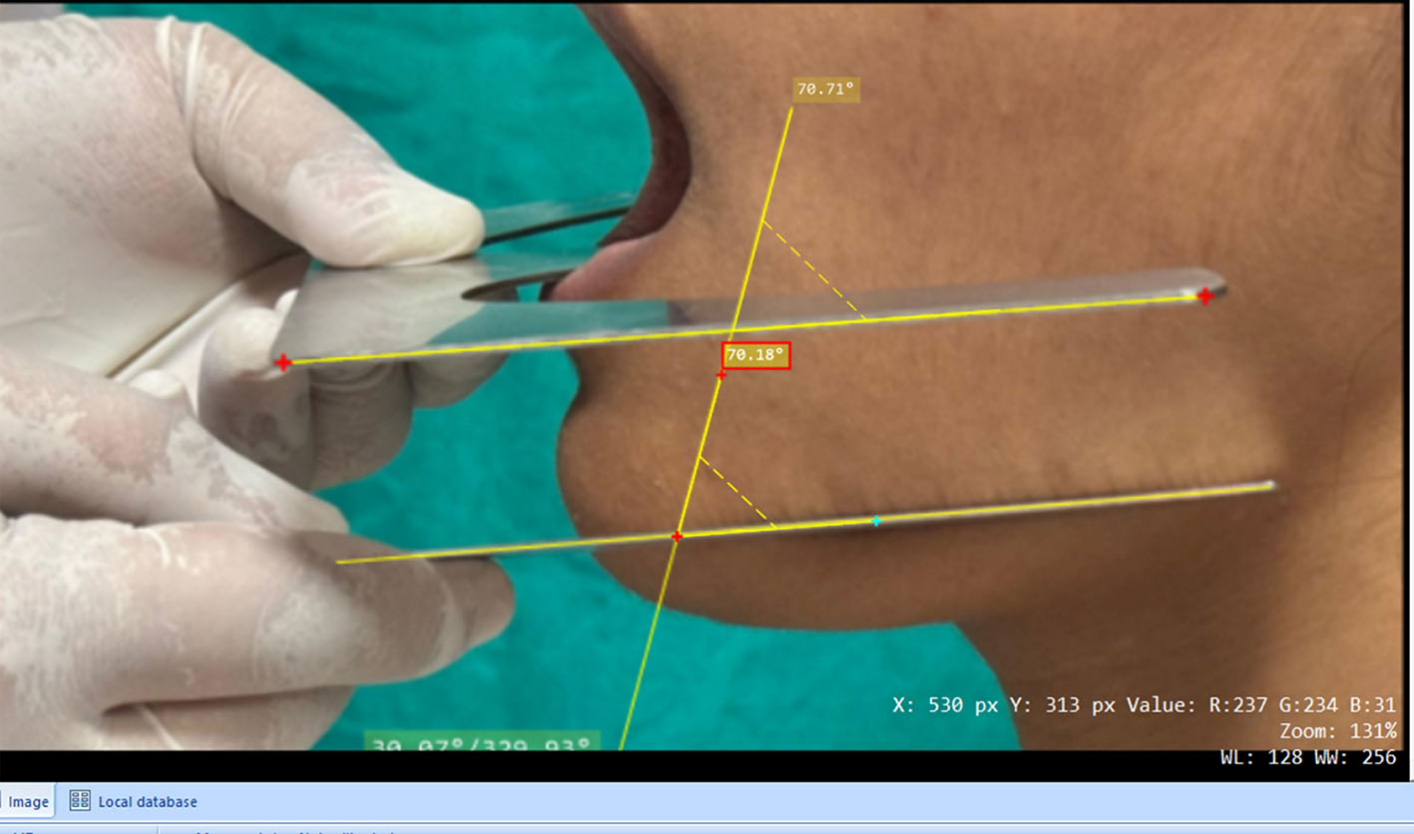
Angle between the occlusal and mandibular planes measured in MicroDicom.

Photographs were taken under standard conditions with a digital camera (Sony digital camera model No. P200 with 3× optical zoom). Photographs were taken in macro mode with a focal length of 20- centimetres, aperture size maximum of f = 8, shutter speed of 1/800, resolution of 7.2 megapixels, sensitivity of ISO 100, compression format of JPEG, and camera height variable (according to the subject’s height). The arms and adjustable plates of the tripod stand were set so that the camera was parallel to the horizontal to get a 1:1 image at 60 meters. Photographs were transferred to Microdicom software, and the parallelism between the mandibular plane and lower occlusal plane was evaluated. ( Figure 3).

The data obtained were evaluated and statistically analysed using the Mann Whitney U test.

## Results

Data was coded in MS Excel and all statistical analysis were carried out using IBM SPSS 27 software. The quantitative variables were presented using maximum, minimum, mean, median, Standard Deviation (SD) and Inter Quartile Range (IQR). Further, the data was checked for normal distribution using the Shapiro- Wilk test. It is observed that the measurement of right and left side does not follow normal distribution. Hence, Non-parametric test (Mann Whitney U test) is used to compare the medians of the two study group.

**Figure 4.**
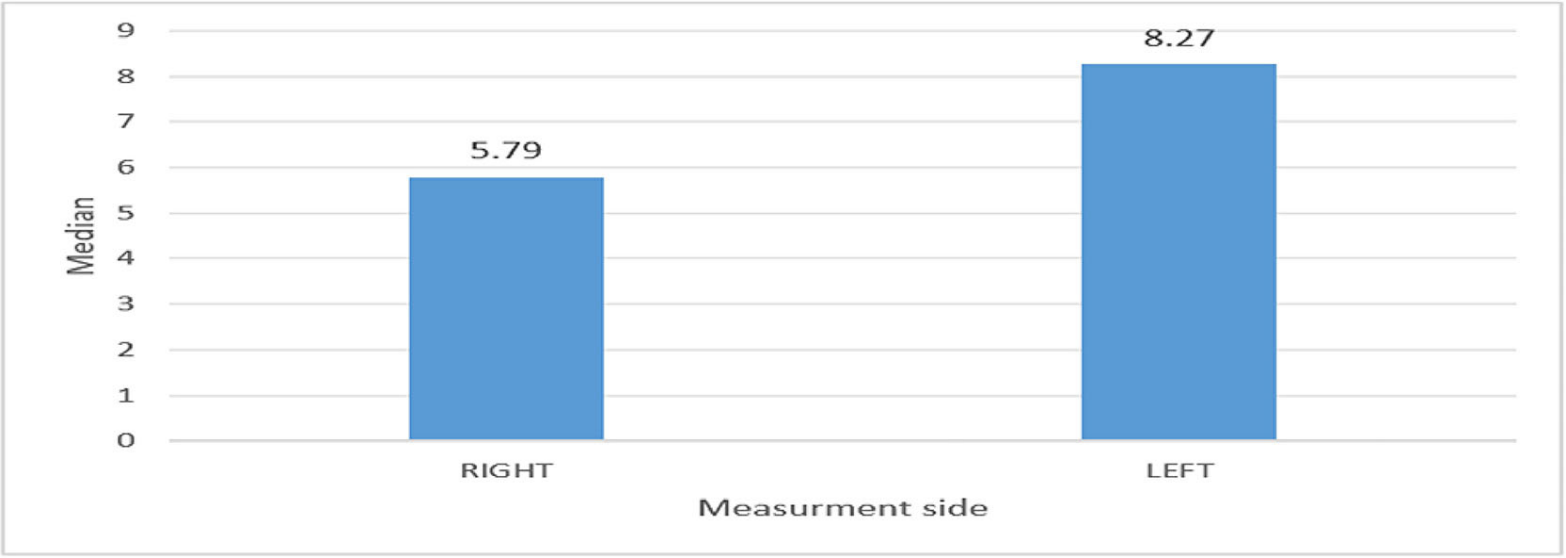
Showing angle between mandibular and lower occlusal plane.

The angle between the mandibular plane and the lower occlusal plane is presented in Figure 4.

On the right side, the mean angle found was 7.03, with a standard deviation of 11.51, and on the left side of the patient, the mean angle found was 6.94, with a standard deviation of 4.13. The mean angle difference between the right and left sides was not statistically significant in Table 1.

**Table 1.** The mean angle difference between the right and left sides.

	Minimum	Maximum	Mean	SD	Median	IQR	Mann Whitney U statistic (p value)
RIGHT	0.0	11.50	7.03	11.51	5.79	6.0	5653.5 (0.110)
LEFT	0.0	14.35	6.94	4.13	8.27	7.50	

## Discussion

In oral rehabilitation, accurate occlusal plane determinations are critical. The occlusal plane’s orientation affects physiologic activities in the oral cavity. The occlusal plane must have the correct height and width for optimal buccolingual exchange and management of food, speech articulation, contacts, tongue space, aesthetics, and buccal soft tissue support. The occlusal table is a milling surface that is deliberately situated in such a way that the tongue on the lingual side and the buccinator muscle on the buccal side retain the food bolus while mastication occurs. This relationship between the tongue and the buccinator muscle will be compromised if the occlusal plane is incorrect. ^3^

The occlusal plane is part of Hanau’s quint for balanced occlusion and one of the factors that can be controlled by the dentist. The posterior level of the occlusal plane is crucial for mandibular function and temporomandibular joint health. TMJ difficulties may occur when the posterior location of the occlusal plane is furthest from the centre of the ramus. According to Boucher, the teeth must be placed in the same location as the original teeth they would be replacing. The vertical height of the occlusal plane in the anterior area is commonly determined by aesthetics and less frequently by functional requirements. ^7^

The Frankfort horizontal plane is the most common reference as it is a reliable anatomical and craniometrical landmark. It has little influence on tooth loss and may be identified easily with a lateral cephalogram. ^8^ When utilising the Fox plane, the most common landmark for determining IOP are the Camper plane and the inter-pupillary line.

The curve of Spee is an arc of a circle that passes through the cusp tips of the mandibular teeth and the condyle. Dr. Lawson Broadrick designed an instrument in the year 1993 that could be utilised with a variety of articulators to identify the appropriate position and orientation for the posterior occlusal scheme once the natural curve of Spee was identified. ^9^ Casts must be mounted in the articulator, followed by the face bow transfer. 4-inch arcs are drawn from the anterior and posterior reference points on the broadricks flag, and the optimal location of the mandibular cusp tips can then be demonstrated by drawing a curve across the lower teeth. When restoring the posterior dentition, teeth (or portions of teeth) that are over-erupted, infra-occluded, rotated, or tipped can be evaluated and modified. Determination of the posterior occlusal plane is done using this method. ^10^

The design of the occlusal plane is strongly influenced by aesthetics and function. Compromise can be attained by adjusting the radius of the curve. A conventional four-inch curve would result in a flat posterior curve in individuals with a retrognathic mandible, creating posterior protrusive interferences. Extrusion of the opposing maxillary teeth would result from such “low” mandibular posteriors. The crown-to-root ratio would be less than optimal if the maxillary posterior teeth were restored to this low occlusal plane. When a class II skeletal association exists, a 3.75-inch radius is more acceptable. In a patient with a class III skeletal, however, a four-inch curve would result in a steep posterior curve, causing significant posterior interference. In this case, a 5-inch radius might be more acceptable. ^11^

When there is attrition or when the canine and second molars are missing, the accuracy of the Broadrick plane is questionable. So, other methods should be employed in conjunction with this approach to precisely establish the IOP.

Craddock et al. found that persons with missing posterior teeth were more likely to have one or more teeth that deviated significantly from the ideal Broadrick curve than those with idea arches. Tooth fracture, improperly shaped restorations, tooth tipping and drifting, and lack of full eruption can all cause deviation for a single tooth, which may or may not be typical of the curve fit for the rest of the quadrant. ^9^

Following posterior tooth loss, the degree to which individual teeth deviate from the Broadrick curve is statistically significant. 26% of patients with posterior tooth loss had a 2 mm or greater deviation from the Broadrick plane, indicating that restoring normal occlusal form and contact is difficult. Orthodontic uprighting of multiple tilted posterior teeth may be suggested to completely restore normal function in terms of tooth loading. Results demonstrating severe deflections of up to 4.55 mm are likely to be clinically significant when restoring posterior occlusal form and function for these patients. ^9^

The lower occlusal plane in the natural dentition extends from the incisal edges of the lower anterior teeth to the points of the posterior teeth’s cusps to a position about two-thirds of the height of the retromolar pad. ^5^ The height of the retromolar pad, which is most used as the posterior reference point, is difficult to locate in dentate individuals with the presence of 2 ^nd^ molars.

According to D’Souza et al., the angle formed radiographically between the mandibular plane and occlusal plane was found to be 13.9 degrees, ^12^ and Sato et al. evaluated the Frankfort occlusal plane angle and mandibular plane and found it to be 14.5 degrees. ^13^

We found a mean of 6.98 degrees between the mandibular plane and the occlusal plane. In our study, it was seen that on the right side, the mean angle found was 7.03, with a standard deviation of 11.51, and on the left side of the patient, the mean angle found was 6.94, with a standard deviation of 4.13. The mean angle difference between the right and left sides was not statistically significant.

The study group had no parallelism between the occlusal plane and the mandibular plane. This finding contrasts with previous research, which has reported an average angulation of approximately 14 degrees between these two planes. In our study, we measured a significantly lower angle of 6.98 degrees.

Several factors could contribute to this discrepancy. One possible explanation is the influence of soft tissue thickness, which can vary among individuals and potentially affect the positioning of the occlusal plane relative to the mandibular plane. Variations in soft tissue may alter the way the dental arches relate to the underlying skeletal structure, thereby impacting the observed angle.

Additionally, the differences could arise from the specific demographic characteristics of our study population compared to those in other studies. Factors such as age, gender, and ethnic background might play a role in these measurements. Further research is warranted to explore these variables and their potential impact on the relationship between the occlusal and mandibular planes.

The limitation of the study is that we did not consider subjects with class 2 and class 3 malocclusion and with different facial forms.

## Conclusion

The mandibular plane and occlusal plane are not parallel to each other, and they form an angle of 6 degrees. Though not parallel, the mandibular plane may give an initial guide in establishing the occlusal plane.

## Ethics and consent

Ethical approval was obtained from Yenepoya Ethics Committee-2, Yenepoya (Deemed to be University) (YEC2/636), and 100 volunteers of 25–30 years of age meeting the inclusion/exclusion criteria were enrolled for the study. The proposed research was explained to them, and written informed consent was obtained.

## Data Availability

Dataset for Evaluating the Relationship Between Mandibular Plane and Lower Occlusal Plane in Fully Dentulous Patients. https://doi.org/10.5281/zenodo.19250122 (Ragher et al., 2026). ^14^ Data are available under the terms of the Creative Commons Attribution 4.0 International license (CC-BY 4.0).
