# Supplementary material for: EVALUATING THE RELATIONSHIP BETWEEN MANDIBULAR PLANE AND LOWER OCCLUSAL PLANE IN FULLY DENTULOUS PATIENTS
Source: F1000Res. 2026 May 12;15:710. [Version 1] doi: 10.12688/f1000research.179633.1 (PMC13216774; doi:10.12688/f1000research.179633.1)
Supplement: Peer review history

# Reviewer report: Reviewer response for version 1

**DOI:** 10.5256/f1000research.198166.r484419

**Published:** 2026-05-27

**Authors:** Praveen Jodalli

**Affiliations:** 1 Manipal Academy of Higher Education, Manipal, Karnataka, India

The introduction is excessively descriptive and may be condensed. Keywords should avoid duplication of words already present in the title. The rationale for selecting only subjects aged 25–30 years should be discussed. The authors can explain why only dentulous subjects with normal occlusion were included

 Ethical approval details are appropriately provided; however, informed consent procedures may be described more clearly. The authors should clarify whether measurements were repeated to reduce observer bias.

 Figure legends should be more descriptive. Recommendations for future research should be added at the end of the discussion section. Improve grammatical structure throughout the manuscript

 The manuscript presents a clinically relevant and scientifically valuable investigation into the relationship between the mandibular plane and lower occlusal plane in fully dentulous patients. The topic is important in the field of prosthodontics, particularly in occlusal rehabilitation, complete denture fabrication, and full-mouth restorative procedures where accurate orientation of the occlusal plane plays a critical role in functional and esthetic outcomes. The study is well organized with clearly defined objectives, appropriate inclusion criteria, and a straightforward methodology. The use of photographic analysis combined with digital software

 measurement provides a practical and non-invasive approach for evaluating occlusal plane orientation. The sample size appears adequate, and the statistical analysis supports the conclusions drawn by the authors.

 The manuscript demonstrates good scientific flow, beginning with a comprehensive introduction that establishes the clinical significance of occlusal plane determination and discusses existing methods used in prosthodontic practice. The discussion appropriately correlates the present findings with previously published literature and highlights the potential clinical applicability of the mandibular plane as an initial guide during occlusal plane establishment.

 The figures and tables effectively support the text, and the conclusions are consistent with the study objectives and results. The study contributes useful clinical information and offers practical relevance for prosthodontists and restorative dentists involved in occlusal rehabilitation procedures.

 Overall, the manuscript is informative, clinically meaningful, and contributes positively to the existing body of prosthodontic literature. It is suitable for indexing.

Is the work clearly and accurately presented and does it cite the current literature?

Yes

If applicable, is the statistical analysis and its interpretation appropriate?

Yes

Are all the source data underlying the results available to ensure full reproducibility?

Yes

Is the study design appropriate and is the work technically sound?

Yes

Are the conclusions drawn adequately supported by the results?

Yes

Are sufficient details of methods and analysis provided to allow replication by others?

Yes

Reviewer Expertise:

Dentistry, cariology, prevention, public health

I confirm that I have read this submission and believe that I have an appropriate level of expertise to confirm that it is of an acceptable scientific standard.

# Reviewer report: Reviewer response for version 1

**DOI:** 10.5256/f1000research.198166.r484420

**Published:** 2026-05-27

**Authors:** Anupama Aradya

**Affiliations:** 1 JSS Dental College and Hospital, Mysore, Karnataka, India

1. The manuscript addresses an important and clinically relevant topic in prosthodontics, particularly in relation to occlusal plane determination during prosthetic rehabilitation.
2. The study methodology is clearly described and appears reproducible, especially the standardized photographic protocol and use of MicroDicom software for angular measurements.
3. The study included only subjects with orthognathic profiles and excluded Class II and Class III malocclusions; therefore, the generalizability of the findings is limited.
4. The authors have provided a detailed literature review discussing conventional methods of occlusal plane determination and the relevance of the Broadrick occlusal plane analyser.
5. The discussion correlates the present findings with previous studies and attempts to explain the differences observed.
6. The manuscript highlights a clinically applicable finding that the mandibular plane may serve as an initial guide for establishing the occlusal plane.
7. The manuscript would benefit from language editing and grammatical revision to improve readability and overall flow of the text
8. Overall, the manuscript contributes useful preliminary information to the field of prosthodontics and craniofacial analysis

Is the work clearly and accurately presented and does it cite the current literature?

Yes

If applicable, is the statistical analysis and its interpretation appropriate?

Yes

Are all the source data underlying the results available to ensure full reproducibility?

Yes

Is the study design appropriate and is the work technically sound?

Yes

Are the conclusions drawn adequately supported by the results?

Yes

Are sufficient details of methods and analysis provided to allow replication by others?

Yes

Reviewer Expertise:

Prosthodontics, dental implants, cement retained implant restorations, gerodontology

I confirm that I have read this submission and believe that I have an appropriate level of expertise to confirm that it is of an acceptable scientific standard.
